# Pattern Recognition Algorithms in Pharmacogenomics and Drug Repurposing—Case Study: Ribavirin and Lopinavir

**DOI:** 10.3390/ph18111649

**Published:** 2025-10-31

**Authors:** Hiram Calvo, Diana Islas-Díaz, Eduardo Hernández-Laureano

**Affiliations:** 1 Centro de Investigación en Computación, Instituto Politécnico Nacional, Mexico City 07738, Mexico; hcalvo@cic.ipn.mx; 2 Ciencias Farmacéuticas, Universidad Autónoma Metropolitana, Unidad Xochimilco, Mexico City 04960, Mexico

**Keywords:** pattern recognition, machine learning, deep learning, pharmacogenomics, drug repurposing, COVID-19, ribavirin, lopinavir

## Abstract

Pattern recognition and machine learning algorithms have become integral to modern drug discovery, offering powerful tools to uncover complex patterns in biomedical data. This article provides a comprehensive review of state-of-the-art pattern recognition techniques—including traditional machine learning (e.g., support vector machines), deep learning approaches, genome-wide association studies (GWAS), and biomarker discovery methods—as applied in pharmacogenomics and computational drug repurposing. We discuss how these methods facilitate the identification of genetic factors that influence drug response, as well as the in silico screening of existing drugs for new therapeutic uses. Two antiviral agents, ribavirin and lopinavir, are examined as extended case studies in the context of COVID-19, illustrating practical applications of pattern recognition algorithms in analyzing pharmacogenomic data and guiding drug repurposing efforts during a pandemic. We highlight successful approaches such as the machine learning-driven prediction of responders and the AI-assisted identification of repurposed drugs (exemplified by the case of baricitinib for COVID-19), alongside current limitations, including data scarcity, model interpretability, and translational gaps. Finally, we outline future directions for integrating multi-omics data, improving algorithmic interpretability, and enhancing the synergy between computational predictions and experimental validation. The insights presented highlight the promising role of pattern recognition algorithms in advancing precision medicine and accelerating drug discovery, while recognizing the challenges that must be addressed to fully realize their potential.

## 1. Introduction

Drug discovery and development is a lengthy, costly, and complex process, traditionally spanning many years for a new therapy to reach patients. Recently, pattern recognition algorithms, an umbrella term encompassing machine learning (ML) and related data-driven techniques, have begun to revolutionize drug discovery by enabling the automated analysis of large and complex biological datasets. These algorithms excel at identifying meaningful patterns and correlations that might elude manual analysis, thus offering powerful support in various stages of drug research. Two areas that have particularly benefited are pharmacogenomics and drug repurposing. Pharmacogenomics investigates how interindividual genetic variation influences drug efficacy and toxicity, providing a foundation for personalized medicine. Pattern recognition techniques offer powerful tools to extract meaningful biomarkers and genetic signatures from complex genomic datasets, enhancing the prediction of drug responses [1,2,3]. In parallel, drug repurposing (also referred to as drug repositioning) explores alternative therapeutic indications for existing compounds—a strategy that gained renewed relevance during the COVID-19 pandemic. In this context, machine learning and network-based methodologies were extensively used to rapidly evaluate thousands of approved drugs for potential activity against novel pathogens [4,5].

The convergence of these fields holds great promise: in a global health crisis like COVID-19, the rapid identification of effective treatments can save lives and considering patient-specific genetic factors can further refine therapy to those most likely to benefit [6]. However, significant challenges remain. Data on pharmacogenomic markers for new diseases (such as COVID-19) are often scarce, and many computational repurposing predictions have yet to translate into clinical efficacy. This points to a need for more advanced algorithms, better integration of heterogeneous data sources, and close coupling of in silico findings with experimental validation.

While the literature on computational pharmacogenomics and AI-assisted repurposing has expanded rapidly, few accounts examine their convergence explicitly through a pattern recognition lens. By integrating advances across language- and graph-based DTI, perturbational signatures, heterogeneous knowledge graphs, and PGx multi-omics, this review seeks to articulate where the evidence is strongest, where translation has failed, and how hybrid pipelines can close the in silico-to-clinic gap [7,8,9,10].

In this paper, we review the current state of the art in pattern recognition algorithms applied to pharmacogenomics and drug repurposing, highlighting key techniques and findings from the recent literature. In particular, we use the examples of ribavirin and lopinavir—two antivirals considered for COVID-19 treatment—as case studies to illustrate how pattern recognition methods can be applied to analyze drug efficacy, pharmacogenomic associations, and repurposing opportunities. We discuss both successful applications (including notable achievements during the COVID-19 pandemic) and the limitations that have been encountered. Finally, we outline future research directions needed to overcome current hurdles, such as incorporating multi-omics data, improving model interpretability, and ensuring that computational predictions robustly inform clinical decision-making.

## 2. Related Work

Pattern recognition algorithms have been increasingly applied across various domains of drug discovery. Below, we summarize related work in two primary areas: (1) pharmacogenomics, where machine learning and statistical pattern recognition methods help uncover genetic determinants of drug response; and (2) drug repurposing, where computational approaches identify existing drugs that might be effective for new indications.

### 2.1. Pattern Recognition in Pharmacogenomics

Early applications of machine learning in pharmacogenomics demonstrated the feasibility of predicting drug response phenotypes from genomic and clinical data. For example, Ke et al. [1] employed classification algorithms including multilayer feed-forward neural networks and support vector machines (SVMs) to model treatment outcomes in chronic hepatitis C patients. In that study, patient data such as genetic polymorphisms (single-nucleotide polymorphisms, SNPs) were used to predict sustained virologic response to combination therapy with interferon-α and ribavirin, achieving promising accuracy in linking SNP patterns to treatment response. Similarly, Lin and Hwang [3] developed an SVM-based approach to assess the efficacy of interferon-α/ribavirin therapy and showed that SVM models could effectively classify responders vs. non-responders using patients’ genetic profiles. Other studies explored artificial neural networks for pharmacogenomic prediction: Lin et al. [2] demonstrated that neural network models can learn complex nonlinear relationships between genomic markers and interferon treatment outcomes. These and related studies established a foundation for applying pattern recognition techniques to pharmacogenomic data, moving beyond traditional statistical analyses.

Beyond hepatitis C, pattern recognition has been applied to pharmacogenomic questions in diverse therapeutic areas. Lin et al. [11] presented pattern recognition techniques combined with haplotype analysis to evaluate drug efficacy, illustrating how integrating linkage disequilibrium information can improve the detection of genetic associations with treatment outcomes. In neuropsychiatry, recent research has embraced machine learning for pharmacogenomics: Lin et al. [12] reviewed machine learning and deep learning applications for antidepressant treatment response in major depressive disorder, highlighting that these methods have shown potential in predicting patient-specific outcomes, identifying genomic and epigenomic biomarkers of response, and even incorporating neuroimaging data to refine predictions. Such studies underscore that modern pattern recognition algorithms (including deep neural networks) can handle high-dimensional data and capture complex interactions among genetic and clinical factors, which is crucial for understanding multifactorial drug response phenotypes.

A cornerstone of pharmacogenomics is genome-wide association studies (GWAS), which scan the genome for variants associated with drug response or toxicity. GWAS themselves can be viewed as pattern recognition approaches in a broad sense, using statistical learning to pinpoint SNPs correlated with phenotypic variation. Indeed, GWAS have yielded important pharmacogenetic findings (for example, identifying *IFNL3/IL28B* gene polymorphisms associated with interferon/ribavirin response in hepatitis C therapy) that later became features in machine learning models [13]. However, GWAS typically focus on single variants and require very large sample sizes to detect associations; machine learning can complement GWAS by building predictive models that aggregate information from multiple variants simultaneously and by incorporating non-genetic features. Recent efforts have combined GWAS with machine learning to improve pharmacogenomic predictions. For instance, Wang et al. [14] performed a GWAS of hepatitis C antiviral therapy outcomes and then used SVM and other classifiers to model treatment success, showing that integrating polygenic risk factors via ML improved predictive performance over considering top GWAS hits alone.

Another area of related work is the use of feature selection and other biomarker discovery techniques in pharmacogenomic datasets. Algorithms such as random forests (which provide variable importance measures) and specialized feature selection methods (e.g., Boruta or minimum redundancy maximum relevance filtering) have been used to isolate the most predictive genetic markers from high-dimensional genomic data [15]. These pattern recognition approaches help researchers focus on key biomarkers (genetic variants, gene expression levels, etc.) that influence drug response. Identifying such biomarkers is essential not only for building accurate predictive models but also for generating biological insights (for example, discovering that variations in drug metabolism genes or immune response genes explain differences in efficacy or toxicity).

Despite the growing body of work, it is noteworthy that pharmacogenomic applications of pattern recognition often face the challenge of limited datasets. Many studies (especially older ones) had sample sizes in the hundreds, which constrained model complexity to avoid overfitting. This has motivated the development of approaches to maximize learning from small-sample data, such as transfer learning or data augmentation in the context of genomic data, though these are still emerging areas. It also underscores the need for collaborative consortia to amass larger pharmacogenomic cohorts. In summary, related work demonstrates that a variety of pattern recognition algorithms—ranging from SVMs and neural networks to statistical GWAS frameworks—have been successfully applied to uncover genotype–phenotype patterns in pharmacogenomics. These efforts have yielded both predictive tools and novel biomarkers, paving the way for more personalized drug therapy.

Beyond classical pattern recognition approaches, recent advances have introduced a new generation of scalable architectures that unify molecular, structural, and network representations for drug discovery. Transformer-style chemical language models such as ChemBERTa leverage masked-language pretraining on tens of millions of SMILES strings to produce generalizable molecular embeddings that enhance downstream property and activity prediction [16]. Similarly, frameworks like DeepPurpose encapsulate sequence- and graph-based encoders for drug–target interaction (DTI) modeling and virtual screening, supporting reproducible benchmarking and rapid repurposing pipelines [17].

At the network scale, foundation graph neural networks (GNNs) trained over large biomedical knowledge graphs have enabled zero-shot inference across thousands of diseases. TxGNN, for example, integrates graph representation learning and metric learning to rank potential indications and contraindications, achieving clinically meaningful performance at the population level [7]. Classic deep frameworks for synergy and compound–protein interaction (CPI/DTI) prediction remain valuable baselines and complementary perspectives: DeepSynergy for drug combination prediction [18], DeepCPI for large-scale compound–protein interaction modeling [19], and multimodal models such as BridgeDPI and DTI-BERT that combine graph and sequence encoders [20,21].

Signature-based inversion methods further connect phenotypic drug effects with disease states. The Connectivity Map (CMap) and LINCS L1000 resource have scaled to over 1.3 million transcriptomic perturbation profiles [8], while the Drug Repurposing Hub provides a curated, mechanism-annotated library for systematic repositioning [22]. Network medicine resources such as Hetionet/Project Rephetio have demonstrated how integrating heterogeneous biomedical relationships yields transparent and testable repurposing hypotheses [9].

Translational caveat. The promise of computational repurposing must be balanced with realistic expectations about clinical translatability. Early COVID-19 predictions like MT-DTI [23] exemplified rapid hypothesis generation; yet randomized evidence later showed a lack of benefits for highly publicized candidates such as hydroxychloroquine and lopinavir/ritonavir in hospitalized patients [24,25,26]. Conversely, baricitinib—surfaced by AI-driven reasoning on host pathways—progressed to positive RCTs when combined with remdesivir [5,27]. These cases underscore that in silico pattern recognition is efficient but must confront biological complexity, experimental uncertainty, and the known translation gap documented across repurposing pipelines [10,28].

Together, these methodological streams—spanning sequence-, structure-, signature-, and network-based perspectives—illustrate how pattern recognition has evolved toward more generalizable and interpretable frameworks. Their integration with pharmacokinetic/pharmacodynamic (PK/PD) and pharmacogenomic (PGx) data promises to strengthen the evidence chain from in silico signals to clinically actionable hypotheses.

### 2.2. Pattern Recognition in Drug Repurposing

Complementing its role in pharmacogenomics, machine learning has also become a vital tool in drug repurposing research. Drug repurposing involves identifying existing drugs that could be effective for new therapeutic indications, often by leveraging extensive biomedical data (such as omics databases, chemical libraries, and clinical records) to find non-obvious drug–disease connections. The urgency of the COVID-19 pandemic in 2020 greatly accelerated research in this area: with limited time to develop new drugs from scratch, scientists worldwide turned to in silico screening and AI to propose candidates among approved drugs that might be re-used to treat COVID-19 [4].

Table 1 contrasts knowledge graph mining, deep DTI/chemogenomics, signature reversal, and executable networks, highlighting typical data inputs and trade-offs.

One class of pattern recognition methods used in repurposing is network-based algorithms. In these approaches, knowledge graphs or networks are constructed, with nodes representing diseases, drugs, genes/proteins, etc., and edges representing known relationships (e.g., a drug targets a protein or a protein is implicated in a disease pathway). Machine learning can then be applied to this network to infer new links, essentially predicting that a drug node should connect to a disease node if certain patterns of connectivity are present. A high-profile example is the work of BenevolentAI during the COVID-19 outbreak: researchers utilized a knowledge graph of human biological processes and pharmacological data to identify existing drugs that could modulate the host pathways exploited by the SARS-CoV-2 virus. This approach led to the prediction that baricitinib, an anti-inflammatory drug used for rheumatoid arthritis, might reduce viral infectivity by inhibiting key host proteins involved in viral entry [5]. Notably, baricitinib was subsequently tested in clinical trials and found to improve outcomes in COVID-19 patients, eventually receiving emergency use authorization [32]. This case illustrates a successful application of AI-driven pattern recognition for drug repurposing: by rapidly analyzing patterns in a vast network of prior knowledge, an existing drug was pinpointed and ultimately proven effective in a completely new context.

**Figure 1:**
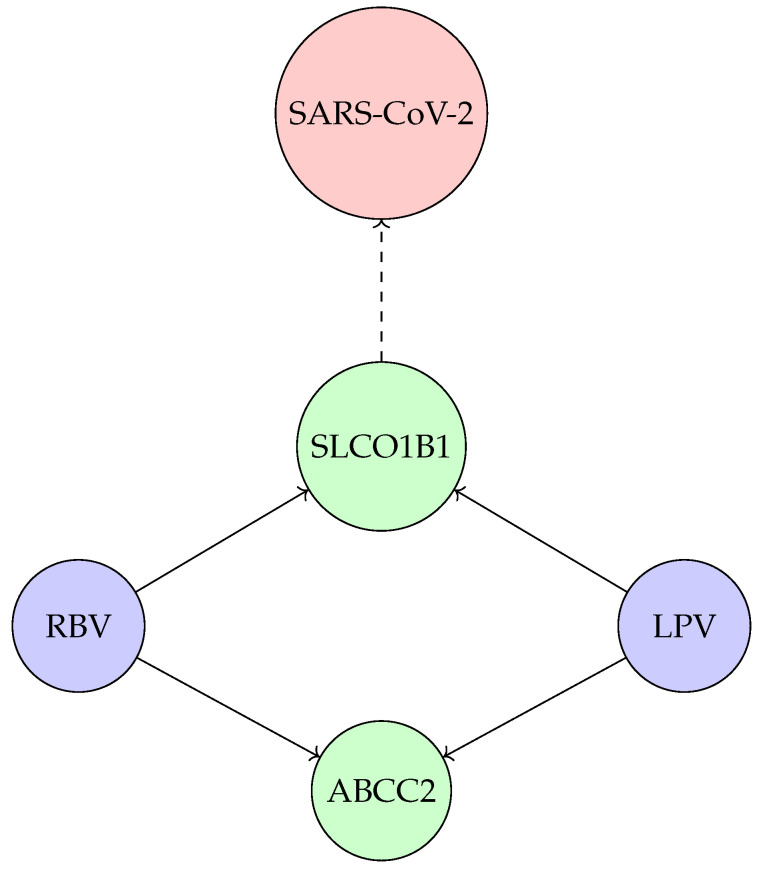
Network of drug–gene–virus relationships highlighting ribavirin (RBV) and lopinavir (LPV) within the context of SARS-CoV-2. Node colors represent biological categories: drugs (blue), genes (green), and viral target (red). Dashed arrows indicate indirect pharmacogenomic connections via gene-mediated drug response.

As an illustration of how drug–gene–virus relationships are abstracted in our framework, Figure 1 summarizes the RBV/LPV context for SARS-CoV-2—Note that this figure offers a conceptual overview of drug–gene–virus interactions in the context of SARS-CoV-2 and is not intended to reflect the full molecular specificity discussed in the case studies. For instance, although RBV is shown connected to SLCO1B1 and ABCC2, the most pharmacogenomically relevant genes for ribavirin are IFNL3 (linked to efficacy) and ITPA (linked to toxicity), as described in detail later. The dashed line connecting SLCO1B1 to SARS-CoV-2 should also be interpreted as an indirect relation: this gene modulates the pharmacokinetics of lopinavir, which was repurposed in response to the viral infection. Thus, the network abstracts complex relationships into a visual shorthand meant to anchor the discussion of case studies rather than serve as a mechanistic diagram.

Another set of techniques involves machine learning models trained on large-scale pharmacological and biomedical datasets to predict drug–disease activity. For instance, deep learning approaches have been developed that take high-dimensional inputs (such as the chemical structure of compounds, gene expression profiles of diseased vs. healthy cells, or signatures of drug perturbation in cell lines) and learn to associate drugs with diseases. Pan et al. [29] (for example) provide guidelines on utilizing deep learning methods for drug repurposing, emphasizing how integrating bioinformatics databases (e.g., gene expression compendia and protein–protein interaction networks) with deep neural networks can accelerate discovery. Common deep learning strategies include autoencoders and other representation learning models that compress complex data (like gene expression changes caused by drugs) and then match these representations to disease signatures, and graph neural networks that operate on molecular or knowledge graph structures to predict new interactions or therapeutic effects. These methods have been applied to identify repurposing candidates in diseases ranging from cancer to neurological disorders, often highlighting candidates that would not have been obvious through traditional reasoning.

During the COVID-19 pandemic, numerous computational repurposing studies employed pattern recognition on different data modalities. Some leveraged high-throughput in vitro screening data combined with machine learning to prioritize compounds for testing. Others, like Zhou et al. [30], used transcriptomic signatures: by comparing the gene expression changes induced by drugs to the gene expression profile of COVID-19 infection in human cells, they used pattern-matching algorithms to find drugs whose effects might counteract the virus-induced changes. Yet other efforts applied systems biology models: Diao et al. [4] discuss a network modeling approach by Howell et al. [33], who built an “executable” network of virus–host interactions and employed a heuristic search (a form of pattern recognition on the network state space) to predict effective combinations of drugs. This model not only generated hypotheses for drug combinations (some of which showed synergistic effects in cell-based assays) but also provided mechanistic explanations for its predictions, highlighting an advantage of network-based pattern recognition in offering interpretable insights.

Related work in this domain also highlights several limitations that constrain the practical impact of computational drug repurposing. Although numerous algorithms can generate candidate lists of repurposed drugs, not all predictions translate into biological efficacy or clinical utility. Early computational suggestions during the COVID-19 pandemic—such as hydroxychloroquine and lopinavir/ritonavir—illustrate this gap. Despite promising in silico evidence and initial enthusiasm, large-scale clinical trials demonstrated no significant benefits for these drugs in hospitalized patients [24,34,35,36]. These outcomes underscore the intrinsic challenges of relying solely on computational predictions, which, while efficient, must grapple with biological complexity, heterogeneous patient responses, and context-dependent pharmacodynamics.

Many computational repurposing pipelines still suffer from high false-positive rates, limited experimental validation, and a lack of reproducibility across datasets [37,38]. The resulting disconnect between computational hypotheses and translational success highlights a critical need for integrative frameworks that combine in silico modeling with experimental feedback loops and pharmacogenomic evidence to bridge the predictive–biological divide.

In response to these shortcomings, researchers have advocated for hybrid approaches that integrate diverse computational techniques. Specifically, combining network-based predictions with structure-based virtual screening and incorporating pharmacogenomic information has been proposed to enhance the reliability and accuracy of therapeutic hypotheses [4]. Another persistent challenge is the quality and representativeness of training data. Pattern recognition models are inherently limited by the biases and gaps in the datasets they learn from; for example, if particular pathways or drug classes are disproportionately represented in reference databases, the resulting models may become biased and overlook less-explored mechanisms of action.

The literature on computational drug repurposing confirms that pattern recognition algorithms, from knowledge graph learning to deep neural networks, can greatly expedite the identification of promising drug candidates for new uses. They have demonstrated successes (as with baricitinib for COVID-19) and provided rich databases of hypotheses for further experimental testing. However, effectively translating these computational findings into clinical impact remains an ongoing effort, underscoring the importance of combining algorithmic predictions with experimental and clinical validation in an iterative loop.

### 2.3. Pattern Recognition in Drug Repurposing and Pharmacogenomics

The application of pattern recognition algorithms, including machine learning (ML) and deep learning (DL), has transformed drug repurposing and pharmacogenomics in recent years. These methods can uncover complex relationships in large-scale biological and chemical data, accelerating the identification of new indications for existing drugs. For instance, Beck et al. [23] employed a transformer-based drug–target interaction model (MT-DTI) to predict antiviral compounds against SARS-CoV-2. Their model, trained on affinity data, screened approximately 3410 approved drugs and highlighted compounds such as atazanavir, remdesivir, and the lopinavir/ritonavir combination (Kaletra) as potential inhibitors of the viral protease, with affinities (Kd) in the nanomolar range. Notably, atazanavir was identified as having the highest predicted potency (Kd ∼ 95 nM), followed by remdesivir (∼113 nM); moreover, lopinavir was predicted to bind to the SARS-CoV-2 replicase with Kd < 1 μM, supporting its early clinical evaluation. This approach demonstrates how DL can recognize subtle patterns in viral protein sequences and drug chemical structures to propose promising repositioning candidates [23].

Another remarkable advancement was introduced by Huang et al. [7], who developed TxGNN, a foundation graph neural network model for zero-shot drug repurposing. TxGNN was trained on an extensive biomedical knowledge graph integrating decades of data (17,080 diseases and ∼7957 drugs). Using metric learning and adaptive feature aggregation, TxGNN can transfer information from diseases with known treatments to those without approved therapies. In large-scale evaluations, TxGNN outperformed conventional models in predicting new indications, improving the area under the precision–recall curve (AUPRC) by up to +19% for indications and +23.9% for contraindications compared to the best baseline. This robust zero-shot performance suggests a novel reasoning ability, further supported by the observation that many TxGNN predictions coincided with off-label prescriptions used by clinicians [7]. Moreover, the model provides interpretability through graph-based reasoning paths, increasing confidence in its findings. Collectively, this work illustrates how pattern recognition on heterogeneous graphs can identify repurposed drug candidates in a multimodal and scalable manner.

Deep attention-based models have also emerged to address complex problems such as combination therapies. Majidifar and Hooshmand [39] proposed two triadic attention-based models—VCTatMLP and VCTatDot—to predict synergistic antiviral drug combinations. Trained on a dataset of viruses, approved drugs, and known interactions, these models learned representations of viruses and compounds, incorporating attention to capture triadic virus–drug–drug interactions. Compared to classical algorithms, one of their attention models and a random forest achieved the best performance in predicting synergistic pairs. Importantly, the AI correctly predicted novel combinations: for instance, acyclovir + ribavirin and acyclovir + inosine pranobex were identified as synergistic against herpesvirus (HSV-1), consistent with previous experimental evidence of enhanced antiviral potency. These results reinforce the validity of the approach, showing high accuracy (relative to traditional methods) and indirect experimental validation of the predictions [39]. Technically, the architectures include a feed-forward neural network variant with attention (attentional MLP) and a customized dot-product attention scheme, suggesting the effectiveness of multivariate attention mechanisms in capturing pharmacological synergy patterns.

In the context of emerging diseases, ML/DL algorithms have been crucial for rapidly identifying candidate therapies. Hashemi et al. [40] proposed a computational framework for antiviral drug discovery against monkeypox virus (MPXV), combining classical ML algorithms (decision trees, SVM, random forests) with a convolutional neural network (CNN) trained on a novel virus–drug dataset. Their platform (MPXV-Pred) integrated similarity measures between MPXV and other viruses, generating viral and pharmacological descriptors for model training. Remarkably, although deep networks typically achieve the best performance, they observed that simpler algorithms could yield comparable accuracy at a lower computational cost. After combining model predictions (voting) and validating via molecular docking, they identified five approved drugs with anti-MPXV potential, including tilorone, valacyclovir, ribavirin, favipiravir, and baloxavir. The inclusion of ribavirin in this list suggests that the learned patterns (e.g., nucleoside analogs active against DNA/RNA viruses) were successfully captured. This study demonstrates an integral workflow where ML/DL pattern recognition, combined with structural simulations, can propose viable repurposing hypotheses in the absence of clinical data.

Additionally, transformer-based models have been explored for pharmacogenomic and drug repurposing tasks. A notable example is TransDTI, a model that employs natural language representations of molecular sequences to predict drug–target interactions [41]. Kalakoti et al. [41] integrated transformer-learned representations (similar to BERT) of proteins and ligands within a drug recommendation pipeline. This approach achieved improvements in interaction classification metrics compared to conventional matrix factorization methods, demonstrating transformers’ capacity to recognize relevant sequential patterns in pharmacogenomics.

In this section, we have shown that the recent literature (2020–2025) presents a wide diversity of architectures: CNNs for chemical/biological data, GNNs for biomedical knowledge graphs, and transformers for biomolecular sequences and language, all converging toward enhancing accuracy (AUC and AUPRC) and predictive power in drug repurposing and pharmacogenomic response prediction. These pattern recognition algorithms have produced novel findings—some experimentally or clinically validated—accelerating progress toward a more personalized, data-driven medicine.

## 3. Methods

In this section, we outline the major categories of pattern recognition and machine learning methods that are employed in pharmacogenomics and drug repurposing. Rather than detailing a specific experimental procedure, we provide a methodological overview suitable for understanding the techniques referenced in our review. These methods include both classical machine learning algorithms and newer advanced approaches, as well as statistical and network-based techniques.

### 3.1. Machine Learning Algorithms for Classification and Regression

A variety of supervised machine learning algorithms have been used to model relationships in pharmacogenomic and drug discovery data. These include linear models (e.g., penalized regression), decision tree ensembles (random forests and gradient boosting machines), and support vector machines (SVMs). In pharmacogenomics, such algorithms are often used in classification tasks, for example, predicting responders vs. non-responders to a therapy based on genotypic and clinical features [1]. SVMs in particular have been popular due to their effectiveness in high-dimensional settings and ability to handle nonlinear decision boundaries via kernel functions. Lin and Hwang [3], as mentioned, utilized an SVM with a radial basis kernel to classify the treatment outcome of hepatitis C therapy, illustrating how SVMs can accommodate complex genotype–phenotype relationships with relatively small sample sizes.

Random forests have also seen extensive use thanks to their robustness and interpretability (via measures like feature importance). For example, a random forest model trained on genetic and biomarker data might rank certain gene variants or expression levels as most important for predicting drug toxicity, thereby highlighting potential biomarkers for follow-up. Machine learning models in drug repurposing are often formulated as regression or ranking problems: a model may predict a continuous score reflecting the likelihood that a given drug will be effective against a disease, which can then be used to rank candidate compounds. These models are trained on known examples of drug–disease pairs (or drug–target interactions) and then generalized to new, untested pairs.

Key to the success of these traditional ML algorithms is careful feature engineering and selection. In genomic data, features can be individual SNP genotypes, allele counts, or aggregated polygenic scores. In chemical data, features might include molecular descriptors or fingerprints. Techniques such as cross-validation and hyperparameter tuning are routinely employed to optimize model performance while avoiding overfitting, especially important given the often limited size of pharmacogenomic datasets. The outputs of these models (predicted probability of response, predicted drug efficacy scores, etc.) can inform decision-making: for instance, a clinician might use a pharmacogenomic classifier to decide whether to prescribe a drug to a patient, or a researcher might prioritize the top-scoring repurposing candidates from a screen for further laboratory testing.

Table 2 summarizes representative pattern recognition models, their inputs, and typical PGx uses (e.g., IFN + RBV response with SVMs).

### 3.2. Deep Learning Techniques

Deep learning refers to the use of neural networks with multiple layers (hence “deep”) that can learn complex, hierarchical representations of data. In recent years, deep learning has made inroads into pharmacogenomics and drug discovery due to its ability to handle large-scale, unstructured data (such as DNA sequences, high-dimensional gene expression profiles, or image data). Convolutional neural networks (CNNs) and recurrent neural networks (RNNs) have been applied to genomic sequence analysis and time-series data, respectively. For example, CNNs have been used to analyze DNA or amino acid sequences for patterns that correlate with drug response—this can be considered a form of pattern recognition on genomic signals. Some studies have even framed viral genome classification as an image/CNN problem by converting genomic sequences to numerical matrices or images [42], although this is more tangential to drug discovery, focusing on pathogen detection.

In the realm of drug repurposing, deep learning models like autoencoders and variational autoencoders have been utilized to learn lower-dimensional representations of drug and disease data that capture essential features. These representations can then be compared or combined to predict novel drug–disease matches [29]. Another promising approach is the use of graph neural networks (GNNs) to operate on networks of molecular interactions or knowledge graphs; GNNs can propagate and aggregate information in a way that uncovers subtle relational patterns. For instance, a GNN can be trained on a network of protein–protein interactions and drug–protein binding data to predict new links, indicating that a drug may affect a disease pathway.

Deep learning has also powered image-based drug discovery and phenotypic screening: high-content imaging of cells treated with various compounds yields rich morphological data, and CNNs can classify or cluster these images to detect which drugs produce “signatures” similar to those of known effective drugs, thus suggesting repurposing opportunities by pattern similarity.

A major advantage of deep learning is its automated feature learning capability—manual feature engineering is reduced, as the network can learn what aspects of the input data are important. However, deep models typically require large training datasets. In pharmacogenomics, where patient numbers might be limited, transfer learning approaches (pretraining a network on a large dataset such as 1000 Genomes or GTEx data, then fine-tuning on a specific smaller pharmacogenomic dataset) can be used to leverage external data. Similarly, in drug repurposing, networks can be pretrained on large chemogenomic databases (like DrugBank or ChEMBL) and then fine-tuned for a specific prediction task.

Sequence-based and graph-based DTI learners achieve competitive affinity classification and regression without 3D structures, leveraging large pretraining on SMILES and protein sequences [16,21]. Frameworks like DeepPurpose provide unified encoders (CNN/RNN/transformer and GNN variants) that ease ablation across encodings and support end-to-end screening workflows from raw sequences to ranked candidates [17]. On heterogeneous biomedical graphs, recent models bridge drug–protein–disease relations with interpretable paths or transformer-based attention over typed edges, improving generalization to rare diseases and under-studied subgraphs [7,20]. Benchmarking against signature-based baselines tied to L1000/CMap [8] and Hetionet-style link prediction [9] clarifies when molecular encoders vs. network context dominate performance and suggests hybrid stacks as a pragmatic route for robust indication transfer.

The interpretability of deep learning remains a challenge. Unlike a decision tree or even an SVM with clear support vectors, neural networks are often considered “black boxes”. Efforts such as attribution methods (e.g., integrated gradients and SHAP values) are increasingly applied to extract some insight from deep models, such as highlighting which genes or pathways are driving a prediction that a certain patient will respond to a drug. This is an active area of research, as interpretability is crucial for clinical adoption of any predictive model.

### 3.3. Genome-Wide Association Studies and Statistical Analysis

While not always termed “pattern recognition” in the classical sense, genome-wide association studies (GWAS) and related statistical analyses form an important methodological pillar in pharmacogenomics. A GWAS involves scanning millions of genetic variants across the genome to find those statistically associated with a drug response phenotype (such as treatment success or occurrence of an adverse drug reaction). GWAS can be seen as recognizing a pattern at the population level: a significant association indicates that a particular genetic variant pattern (e.g., the presence of allele T at a certain locus) correlates with the phenotype of interest.

The output of GWAS is typically a set of candidate variants that show significant associations. These can then be fed into machine learning models as features or used to stratify patients. For example, GWAS identified variants in the *SLCO1B1* gene that predispose patients to simvastatin-induced myopathy; this information is now used clinically to adjust simvastatin doses based on patient genotype. In our context, GWAS of COVID-19 drug responses have been limited (given the lack of standardized treatment in early stages), but by extrapolation from other diseases, one can infer relevant variants. Indeed, experts have pointed out that, although direct pharmacogenetic data for COVID-19 therapies were initially sparse, many implicated drugs have known pharmacogenomic markers in other diseases [6]. GWAS and candidate-gene studies from HIV or hepatitis treatments, for instance, identified variants in metabolizing enzymes and transporters (like CYP3A4, ABCC2, SLCO1B1) that could analogously affect the pharmacokinetics of repurposed antivirals for COVID-19.

Statistical pattern recognition techniques also encompass haplotype analysis and the detection of epistasis (i.e., gene–gene interactions), which extend the traditional scope of genome-wide association studies (GWAS) by incorporating combinations of genetic variants rather than individual SNPs alone. For example, Lin et al. [11] employed haplotype pattern analysis to improve the prediction of drug response, demonstrating that specific combinations of SNPs—haplotypes—may exert stronger phenotypic effects than isolated variants. This resonates with the core idea of pattern recognition: the “pattern” here is a multiallelic genetic signature correlated with a trait of interest.

In pharmacogenomics (PGx), machine learning supports dose optimization and adverse event risk prediction by modeling nonlinear interactions among variants, expression, and clinical covariates. Contemporary reviews synthesize how NGS-era PGx and ML converge in oncology and beyond, emphasizing multi-omics integration and external validity [43,44]. Deep learning for multi-omics data—genome, transcriptome, proteome and metabolome—improves representation of drug response phenotypes and helps bridge the genotype–to–phenotype gap [45,46]. From a systems perspective, network-based multi-omics integration helps manage heterogeneity and missingness while maintaining biological interpretability [47]. These lines of work collectively motivate PGx-aware repurposing workflows in which in silico candidates are stress-tested against PGx priors, predicted PK/PD variability, and population-specific allele frequencies before advancing to experimental validation.

Detecting epistatic interactions is particularly computationally intensive due to the combinatorial explosion of the search space. However, machine learning approaches such as multifactor dimensionality reduction (MDR) and neural networks have been explored to uncover non-additive interaction patterns that conventional GWAS methodologies are likely to overlook.

### 3.4. Biomarker Identification and Feature Selection

Feature selection is a central component of the pattern recognition arsenal, particularly in domains such as pharmacogenomics, where the number of candidate predictors (e.g., genetic variants or gene expression levels) often vastly exceeds the number of available samples. In this context, feature selection itself can be framed as a pattern recognition problem: identifying the most informative subset of variables that explain or predict the phenotype or treatment response.

Sparse regression techniques such as LASSO (L1-penalized regression) perform implicit feature selection by shrinking the coefficients of less relevant variables to zero, thereby retaining only the most salient predictors. Similarly, decision tree-based models inherently prioritize the most informative features by splitting on variables that maximize discriminatory power at each node. These methods help reduce dimensionality, improve generalization, and reveal potential biomarkers for downstream validation.

In recent studies, specific algorithms have been developed for biomarker discovery in high-dimensional data. For instance, the Boruta algorithm (built on random forests) iteratively removes features proven by permutation to be less important than random probes, yielding a confident set of relevant features. In the context of COVID-19, Li et al. [15] applied Boruta and minimum redundancy–maximum relevance (mRMR) filtering to identify key immune cell markers that distinguish COVID-19 from other conditions, which is analogous to identifying disease-specific biomarkers. Although that example is about diagnostic markers, the same techniques could be applied to find predictive biomarkers for drug response (e.g., which cytokine levels or which gene expression changes best predict a good response to an antiviral).

Another emerging concept is the use of pattern recognition to integrate multi-omics biomarkers. Rather than selecting features from a single data type, advanced methods use dimensionality reduction or multi-view learning to combine patterns across genomics, transcriptomics, proteomics, etc. For example, a patient’s genomic variant pattern might indicate something about drug metabolism capacity, while their baseline transcriptomic profile might indicate immune response vigor; combining both via integrative algorithms (such as matrix factorization or deep multimodal networks) can produce a more robust predictor of drug outcome than either alone. These methods inherently perform feature extraction/selection in a multi-omics space, aiming to distill a cross-modal pattern (or set of biomarkers) that drives differential drug response.

### 3.5. Network-Based and Integrative Approaches

Network-based methods were highlighted in the related work for their application in drug repurposing; here, we outline the general approach. Biological and pharmacological knowledge can be represented in networks: for instance, a network could have nodes for genes, drugs, and diseases, with edges encoding relationships (gene–gene interactions, drug–gene targeting, gene–disease associations, etc.). Pattern recognition on networks often involves algorithms that find clusters, paths, or subnetworks that are enriched for a particular property. One example is network propagation algorithms, which simulate a “spread” of influence from a seed node and can identify nodes closely connected in the network. In repurposing, if one takes the known drug targets of a compound as seed nodes in a protein–protein interaction network and propagates influence, one can see which disease-associated genes get “lit up” by proximity, suggesting the drug could impact that disease pathway.

Machine learning can be applied to networks via graph embeddings: methods like node2vec or graph convolutional networks create vector representations of each node capturing the network context. Then, a drug node and a disease node’s vectors can be used in a classifier or predictor to estimate the likelihood of an edge (meaning the drug treats the disease). Such an approach was implicitly used by Richardson et al. [5], where the knowledge graph embedding and mining pointed to baricitinib as a likely COVID-19 treatment by identifying an indirect path: baricitinib → AAK1 kinase (known target) and AAK1 → SARS-CoV-2 endocytosis (virus needs this host factor), thus baricitinib → virus entry inhibition.

Integrative approaches combine heterogeneous data modalities and algorithmic frameworks to generate more robust predictions. For instance, one might link a deep learning model trained to predict drug–target binding affinities with a second model estimating disease–gene associations, thereby enabling the inference of novel drug–disease relationships through learned intermediate representations. In pharmacogenomics, such integration may involve the confluence of genomic variant data, clinical phenotypes, and pharmacokinetic/pharmacodynamic (PK/PD) parameters.

Pandi et al. [48] introduced a machine learning framework designed to evaluate the functional impact of pharmacogenomic variants by integrating a wide array of annotation features. Their model assigns scores to variants, prioritizing those most likely to influence drug response. These tools offer a practical means of filtering the deluge of data generated by high-throughput genome sequencing—such as that obtained from large patient cohorts—down to a shortlist of variants that are computationally predicted to be functionally relevant. Pattern recognition, in this context, serves as a triage mechanism, focusing downstream analyses on the most promising genetic signals.

In summary, the methodological arsenal available for pattern recognition in drug discovery is both broad and diverse. Classical machine learning models remain well-suited for structured datasets with modest sample sizes. Deep learning architectures, by contrast, are capable of capturing high-order nonlinearities in large-scale multimodal datasets. Statistical GWAS and association-based methods provide genome-wide screening tools, while feature selection techniques distill high-dimensional data into tractable biomarker sets. Network-based methods, including graph inference and knowledge graph mining, further enrich this toolkit by incorporating prior knowledge and relational structure.

In practice, many studies adopt hybrid pipelines. A typical workflow might begin with GWAS-based filtering to identify candidate variants, followed by machine learning to predict outcomes of interest. Network analysis may then contextualize these variants within biological pathways, while deep learning models could incorporate additional data modalities—such as transcriptomics or chemical structure—to refine the predictions.

**Figure 2:**
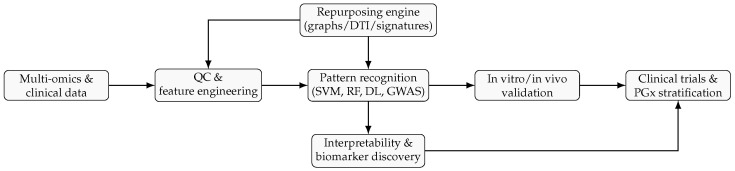
End-to-end AI-assisted pipeline for pharmacogenomics and drug repurposing.

Figure 2 outlines the end-to-end AI-assisted pipeline we reference throughout (data → features → pattern recognition → validation → clinical translation).

In the next section, we demonstrate how these various methodologies converge in two concrete case studies: the repositioning and pharmacogenomic characterization of ribavirin and lopinavir in the context of COVID-19.

## 4. Results and Discussion

**Figure 3:**
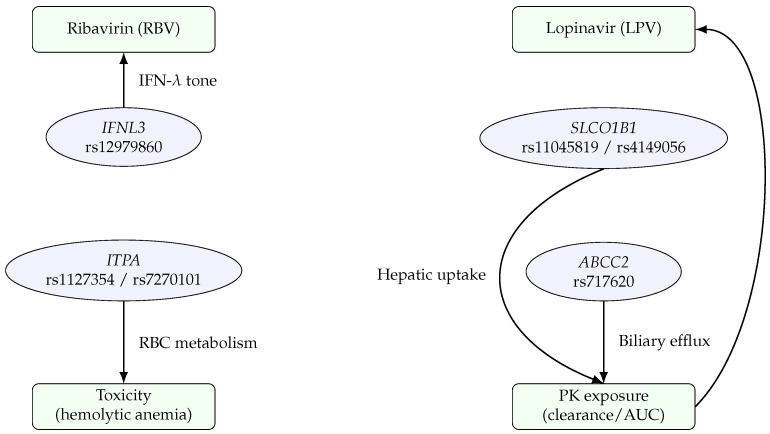
Gene–drug interaction diagram highlighting pharmacogenomic determinants of RBV and LPV pharmacokinetics and pharmacodynamics. Green boxes indicate pharmacological entities (drugs or outcomes), while blue ellipses represent genes or genetic variants.

We now turn to a focused discussion on two case studies, ribavirin and lopinavir, highlighting how pattern recognition approaches have been utilized or could be utilized to glean insights in their use against COVID-19. Ribavirin and lopinavir were among the existing antiviral drugs considered for repurposing during the early stages of the COVID-19 pandemic. Neither drug ultimately became part of the standard of care for COVID-19 due to limited or inconsistent clinical efficacy, but significant effort was invested in studying them. These efforts included analyses of pharmacogenomic markers that might influence treatment response and in silico screenings to predict their effectiveness. By examining these case studies, we illustrate successes, limitations, and lessons learned for pattern recognition in drug discovery. Figure 3 organizes the key pharmacogenomic determinants we discuss for RBV and LPV, separating PD and PK axes. Specific loci discussed in this section are listed in Table 3, with reported effects on toxicity and exposure.

### 4.1. Case Study 1: Ribavirin Pharmacogenomics in COVID-19

Ribavirin is a broad-spectrum antiviral nucleoside analog long used in combination with interferon-α for the treatment of hepatitis C virus (HCV) infection. Its potential application to COVID-19 attracted interest because of its established activity against other RNA viruses. From a pharmacogenomic perspective, ribavirin exhibits both variable efficacy and substantial toxicity—most notably hemolytic anemia—during HCV therapy, and these outcomes have been linked to specific genetic polymorphisms. Pattern recognition approaches originally developed in the HCV context can therefore inform hypotheses about ribavirin’s performance in COVID-19.

**Figure 4:**
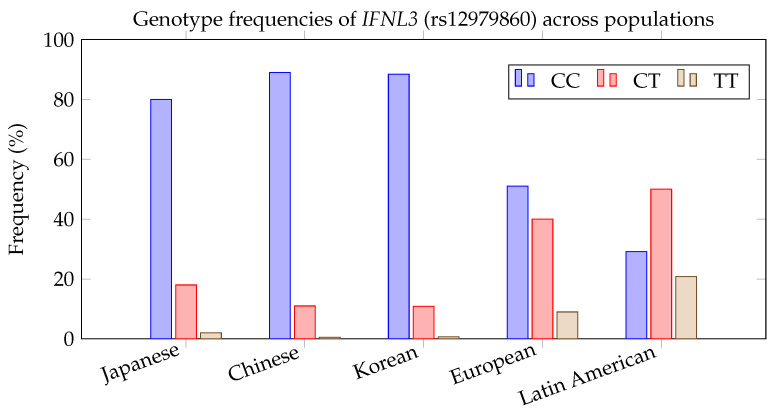
Observed/derived genotype frequencies of *IFNL3* rs12979860 by population. Japanese and Chinese values were derived from 1000 Genomes minor-allele frequencies (T) under Hardy–Weinberg equilibrium (Japan T ≈ 0.10; China T ≈ 0.06). Korean values are observed in health-check examinees. The European point reflects the 1000G EUR average consistent with non-Italian Europeans and CEU, and the Latin American point uses a control population from Buenos Aires as a practical AMR proxy. Sources: 1000 Genomes-based summaries and MAFs for Japanese and Chinese populations; genotype table for continental groups; Korean genotype distribution; Buenos Aires controls [53,54,55,56].

Among the most intensively studied genetic determinants of ribavirin response is a single-nucleotide polymorphism (SNP) in the *IFNL3* gene (also known as *IL28B*). A variant located upstream of *IFNL3* (rs12979860, C/T) became widely known for its association with interferon+ribavirin response in HCV: patients carrying the *CC* genotype achieved significantly higher cure rates than those with CT or TT genotypes [13]. This relationship was first detected through GWAS and later corroborated by independent analyses. Mechanistically, the *CC* genotype is thought to confer a more robust endogenous interferon-λ response. By analogy, it has been hypothesized that the same genetic marker could modulate outcomes if ribavirin were used against SARS-CoV-2, since interferon pathways are central to host antiviral defense. Indeed, Syedbasha and Egli [49] reported that carriers of the *IFNL3* rs12979860 *CT/TT* genotypes exhibit impaired interferon-λ production and poorer responses to antiviral therapy in HCV, suggesting potential for a similarly diminished benefit from ribavirin-based regimens in COVID-19. Pattern recognition models such as classification trees could directly leverage this feature—for example, by splitting on the *IFNL3* genotype as a primary determinant of predicted treatment success. Population-level differences for rs12979860 are illustrated in Figure 4.

A second key pharmacogenomic factor is variation in the *ITPA* gene, which encodes inosine triphosphatase. Variants such as rs1127354 and rs7270101 produce an enzyme deficiency that paradoxically *protects* against ribavirin-induced hemolytic anemia. Multiple studies, including the meta-analysis by Pineda-Tenor et al. [51], have shown that patients with these *ITPA* variants experience attenuated hemoglobin declines during ribavirin therapy, presumably because the deficiency prevents toxic metabolite accumulation in red blood cells. However, as noted by Thompson et al. [13], while these variants strongly mitigate anemia, they do not necessarily increase the likelihood of virologic cure or obviate the need for dose modification in practice. This underscores a broader limitation of pattern recognition approaches focused narrowly on a single endpoint: a model may correctly predict low adverse event risk due to a genetic variant, but clinical decision-making must still weigh efficacy and may not translate such findings into obvious management changes (e.g., physicians might not escalate ribavirin dosage despite a lower predicted anemia risk).

For COVID-19 specifically, no clinical trials to date have incorporated pharmacogenetic stratification for ribavirin; its evaluation was largely confined to small or uncontrolled studies. This means that we lack direct validation whether, say, *IFNL3* or *ITPA* genotypes modulate ribavirin’s effectiveness or safety in SARS-CoV-2 infection. Pattern recognition algorithms can nonetheless be used in a post hoc manner: for example, if genomic DNA from COVID-19 trial participants were available, one could use a regression model to see if the *IFNL3* genotype correlates with viral clearance time or if the *ITPA* genotype correlates with hemoglobin drop, controlling for other factors. In silico, one could also employ a random forest or SVM on an HCV dataset (where outcomes are known) and then test whether the model’s learned importance of these variants holds true in a COVID-19 cohort.

An important observation is that, to date, ribavirin pharmacogenomics for COVID-19 remains largely speculative and extrapolated from HCV data. As noted by Biswas et al. [6], direct pharmacogenetic evidence in COVID-19 is scarce because such studies were not prioritized during the emergency; however, by analogy to other diseases, it is likely that the same genetic variants (e.g., in *IFNL3*, *ITPA*, and others like *SLC28A2* encoding a ribavirin transporter) would have significant roles if ribavirin were widely used in COVID-19. Pattern recognition methods are thus invaluable for two reasons here: (1) they allow us to rapidly combine prior knowledge from different sources (HCV treatment data, molecular biology of interferon, etc.) to predict what factors matter for COVID-19, and (2) they can generate hypotheses (e.g., “IFNL3 genotype will predict COVID-19 ribavirin response”) that can be tested if data become available.

### 4.2. Ribavirin: Pharmacogenomic Findings and Repurposing Beyond HCV

Ribavirin is a broad-spectrum nucleoside analog traditionally used for hepatitis C virus (HCV) infection in combination with interferon. Since 2020, its potential in other viral contexts has been investigated, and its pharmacogenomic determinants have been reviewed across different populations. During the COVID-19 pandemic, ribavirin was considered for therapeutic repurposing due to its prior history against RNA viruses (it had previously been employed against SARS-CoV-1 and MERS-CoV). Although the clinical efficacy of ribavirin in COVID-19 proved limited in trials, these efforts motivated an examination of genetic factors that could influence its pharmacokinetics (PK) and toxicity outside the HCV context [57]. A key pharmacogenomic finding concerns the role of nucleoside transporters: polymorphisms in the *SLC29A1*, *SLC28A2*, and *SLC28A3* genes—encoding equilibrative and concentrative nucleoside transporters—may alter ribavirin uptake and distribution. In particular, patients carrying certain *SLC29A1* variants (e.g., reduced-function alleles of the ENT1 transporter) have been reported to exhibit plasma ribavirin concentrations approximately 10–15% higher than wild-type individuals under equivalent dosing conditions. An association study indicated that homozygotes for one *SLC29A1* variant reached trough levels of ∼2070 ng/mL vs. ∼1837 ng/mL in noncarriers, a significant difference that could increase the risk of hemolytic anemia. These data demonstrate that genetic variations in uptake transporters modulate systemic exposure to ribavirin, a factor that should be considered when extrapolating its use to new indications or populations [6].

Beyond PK, the hematologic toxicity of ribavirin (hemolytic anemia) is strongly influenced by genetics. Multiple studies have confirmed the association between polymorphisms in the *ITPA* gene (inosine triphosphatase) and protection against ribavirin-induced anemia. A common defective allele of *ITPA* leads to reduced enzymatic activity, which paradoxically mitigates the hemoglobin drop under ribavirin treatment by limiting the accumulation of toxic inosine metabolites [57]. A meta-analysis of 20 studies (in HCV patients) showed that carriers of loss-of-function *ITPA* alleles experienced a significantly smaller decrease in hemoglobin (∼1.0 g/dL less reduction on average) compared to wild-type individuals during ribavirin therapy. Although these data primarily derive from the HCV context, similar findings extend to short-term ribavirin use in other infections: for example, hemolytic anemia has been reported in patients treated with ribavirin for respiratory syncytial virus (RSV) infection after transplantation, suggesting that the adverse effect and its pharmacogenetic basis (*ITPA*) are not exclusive to HCV. Overall, *ITPA* emerges as a validated pharmacogenomic marker to anticipate ribavirin tolerability regardless of indication.

Regarding new applications investigated since 2020, in silico and preclinical studies have explored ribavirin’s potential against emerging viral infections. On one hand, the aforementioned repurposing strategy for monkeypox virus (MPXV) identified ribavirin among the top candidate compounds. The MPXV-Pred model by Hashemi et al. [40] prioritized ribavirin due to viral similarity patterns, yielding high prediction scores that led to its inclusion among final candidates. Molecular docking studies indicated that ribavirin can interact favorably with the viral RNA polymerase of MPXV, supporting its potential mechanism of action in this emerging infection. Although experimental confirmation against MPXV is still pending, this result suggests that ribavirin’s antiviral signature—viral polymerase inhibition—is computationally recognized as relevant beyond classical RNA viruses. Similarly, Majidifar and Hooshmand [39] found that ribavirin could potentiate acyclovir activity against herpesvirus (HSV-1) in synergistic combinations. This prediction aligns with independent reports showing additive or synergistic effects of ribavirin combined with nucleoside analogs against herpesvirus, possibly through exacerbation of viral nucleoside stress. Although the clinical use of ribavirin against herpesvirus remains unestablished, such findings broaden the theoretical spectrum of its potential repurposing.

Another recent line of research evaluated ribavirin in COVID-19 combination regimens. For instance, in early clinical studies, ribavirin was administered together with lopinavir/ritonavir and interferon in patients with severe COVID-19; while the results did not show a strong benefit over controls, they provided valuable safety data. Takahashi et al. [57] reviewed the pharmacogenomic literature in COVID-19 and proposed that, should ribavirin use be extended to this disease, its known genetic markers (e.g., *ITPA* and transporter variants) should be considered to maximize safety. Since ribavirin may cause hemolysis and bone marrow suppression, in critically ill COVID-19 patients, it was emphasized that therapy should be individualized based on genotype to prevent complications in genetically susceptible individuals. To date, no GWAS studies outside the HCV context have identified novel variants modulating ribavirin efficacy, likely due to its limited use beyond viral hepatitis. Nevertheless, a previous GWAS in HCV patients (*n* = 303, Thai population) identified variants in *DDRGK1*, in addition to *ITPA*, associated with thrombocytopenia induced by ribavirin and interferon. This suggests that other genes (e.g., those involved in hematologic toxicity) could influence ribavirin response and merit investigation if the drug is repurposed in new clinical scenarios.

In summary, ribavirin has been the subject of intensive pharmacogenomic analyses, delineating a clear profile: polymorphisms in *SLC28/29* transporters affect its pharmacokinetics, and variants in *ITPA* largely determine its hematologic toxicity. The extrapolation of these findings to contexts beyond HCV (such as COVID-19 or other viral infections) is supported by current evidence [6,57]. While ribavirin’s new therapeutic applications remain limited, recent computational and preclinical studies have suggested its potential utility against emerging pathogens, offering hypotheses that warrant experimental validation. Any repurposed use of ribavirin should be accompanied by pharmacogenomic patient stratification to optimize dosing and minimize risks—an example of how integrating machine learning and pharmacogenomics can guide personalized medicine in antiviral therapy.

The case of ribavirin illustrates both the power and limitations of pattern recognition in pharmacogenomics. On one hand, algorithms helped identify clear genetic patterns (*IFNL3* and *ITPA* variants) that affect drug response, offering a route to personalize therapy. On the other hand, translating those patterns to a new disease context (COVID-19) is not straightforward without data, and even the known patterns address only part of the therapeutic equation (e.g., toxicity vs. efficacy trade-offs). As a successful approach, one could envision a future study where COVID-19 patients indicated for ribavirin (perhaps in a new outbreak scenario) are stratified by a machine learning model that incorporates their genomic data to guide dosing or concomitant therapy. The current limitation is a lack of such integrative clinical datasets; thus, one of the future directions is to systematically collect genetic data in clinical trials and apply pattern recognition retrospectively to inform subsequent trials.

### 4.3. Case Study 2: Lopinavir Pharmacogenomics and Machine Learning Repurposing

Lopinavir, co-formulated with ritonavir (as Kaletra^®^), is an HIV-1 protease inhibitor that attracted attention early in the COVID-19 pandemic as a candidate for antiviral repurposing. Although large clinical trials such as RECOVERY ultimately found no clinical benefit in hospitalized COVID-19 patients, the drug remains a valuable case for exploring how pharmacogenomic variability and computational modeling intersect in drug repositioning.

From a pharmacogenomic standpoint, several polymorphisms influence lopinavir disposition and response. Lubomirov et al. [52] conducted a two-stage pharmacogenetic analysis in HIV-infected individuals and identified variants in the hepatic transporter gene *SLCO1B1* with opposing functional effects: rs11045819 (SLCO1B1*4) was associated with increased clearance, whereas rs4149056 (SLCO1B1*5) correlated with reduced clearance. A variant in *ABCC2* (encoding efflux transporter MRP2) and another tagging the *CYP3A* cluster were also linked to clearance differences. These genetic effects produced inter-patient variability exceeding threefold in lopinavir plasma concentrations at identical doses [52]. Pattern recognition models could incorporate these features to predict drug exposure: for example, linear regression could estimate AUC as a function of genotype or a decision tree could classify patients as likely “rapid” or “slow” metabolizers based on combined transporter and enzyme profiles.

During COVID-19, one explanation for lopinavir’s poor efficacy was its inability to reach inhibitory concentrations in pulmonary tissue. Repurposing would therefore benefit from identifying patient subgroups predisposed to higher systemic levels—e.g., carriers of the SLCO1B1*5 allele associated with slower clearance. Conversely, individuals carrying multiple “high-clearance” variants could be predicted to under-dose unless therapeutic drug monitoring or dose adjustments were applied. Pattern recognition workflows (such as logistic regression or SVM classifiers) could be used to estimate the probability of achieving effective plasma levels based on genotype, demographic, and co-medication data, allowing stratified trial designs in future outbreaks.

Another avenue involves machine learning assessment of variants of uncertain significance. Pandi et al. [48] proposed a classifier integrating evolutionary conservation, protein-structure features, and clinical annotations to predict whether pharmacogenomic variants alter drug response. Such a framework could evaluate rare variants in *SLCO1B1*, *ABCC2*, or *CYP3A4/5* relevant to lopinavir metabolism, nominating candidates for further validation even when empirical data are lacking. This highlights how pattern recognition techniques extend beyond known polymorphisms, supporting the interpretation of novel genomic data as they emerge.

Beyond host genetics, computational algorithms were deployed to predict whether lopinavir itself would inhibit SARS-CoV-2. Early in silico studies suggested binding of lopinavir to the viral main protease (3CL_pro_), yet these results relied on static docking. A more integrative approach by Beck et al. [23] applied a deep learning drug–target interaction (MT-DTI) model trained on antiviral compounds to rank potential therapies. Although lopinavir/ritonavir appeared among plausible hits, subsequent validation revealed that atazanavir and remdesivir exhibited stronger predicted affinities. The clinical failures underscored the importance of integrating pharmacokinetic and pharmacodynamic constraints—variables that many pattern recognition models still overlook. Nevertheless, expert-guided data mining by Biswas et al. [6] converged on a consistent pattern linking lopinavir with transporter genes (*SLCO1B3*, *ABCC2*), emphasizing that human-curated pattern recognition remains valuable when training data for automated models are sparse.

In summary, the lopinavir case in COVID-19 illustrates how computational prediction and pharmacogenomic insight must be combined. Machine learning can identify genetic determinants of exposure and model potential antiviral activity, but translational success requires incorporating tissue distribution, host factors, and real-world pharmacology into the pattern recognition framework.

### 4.4. Lopinavir: Pharmacogenomic Interactions and Antiviral Efficacy

Beyond the COVID-19 context, lopinavir’s pharmacogenomic landscape and repurposing potential continue to be explored across viral diseases. Interindividual differences in pharmacokinetics are largely driven by polymorphisms in hepatic transporters and conjugation pathways. Variants in *SLCO1B1*/*SLCO1B3* (OATP1B1/B3) and *ABCC2* (MRP2) alter lopinavir disposition by modifying hepatic uptake and biliary efflux [6]. The reduced-function *SLCO1B1* c.521T>C (Val174Ala) allele, known to decrease OATP1B1 activity, produces higher plasma exposure—up to ∼40% greater AUC in some cohorts—potentially increasing gastrointestinal or hepatic adverse events. Similarly, certain *ABCC2* variants limiting efflux have been associated with elevated systemic levels. While ritonavir’s potent CYP3A4 inhibition buffers metabolic variability, subtle differences in *CYP3A5* expression may still influence clearance [57]. Although genotype-based dosing guidelines are not yet established, these findings reinforce the clinical relevance of transporter polymorphisms in lopinavir pharmacokinetics.

Additional host variants may influence tolerability. Biswas et al. [6] noted that the HLA-B*57:01 allele—classically linked to abacavir hypersensitivity—could merit evaluation, even though no immunologic reactions have been documented with lopinavir/ritonavir. Likewise, carriers of *UGT1A1* polymorphisms can show mild bilirubin elevations, as observed with atazanavir. From a clinical standpoint, such markers may inform safety monitoring rather than efficacy adjustments.

Lopinavir has also been investigated in repurposing efforts beyond HIV and SARS-CoV-2. Patel et al. [58] employed AI-driven molecular dynamics to identify HIV protease inhibitors—including lopinavir—as potential modulators of monkeypox virus (MPXV) proteases, while other reports have explored its possible use in BK polyomavirus infection following renal transplantation. These findings remain preliminary but demonstrate ongoing interest in repositioning protease inhibitors across viral families. The recurring computational identification of lopinavir, despite limited clinical success, highlights both the promise and the pitfalls of data-driven repurposing: high predicted binding affinity does not guarantee adequate bioavailability or therapeutic index.

From a practical pharmacogenomic viewpoint, interactions with concomitant medications are crucial. The *SLCO1B1* c.521T>C variant that elevates lopinavir exposure also increases statin-related myopathy risk; thus, the co-administration of lopinavir/ritonavir with statins in variant carriers may heighten combined toxicity. Integrating pharmacogenetics with polypharmacy management is therefore essential in repurposing scenarios. As Biswas et al. [6] and others argue, future implementations of lopinavir/ritonavir should include pre-treatment genotyping for *SLCO1B1/B3* and *ABCC2* to optimize dosing or select alternative therapies for atypical metabolizers.

Overall, lopinavir exemplifies how pharmacogenomic diversity and computational modeling converge in the modern understanding of antiviral therapy. Genetic variation in transporters (*SLCO*/*ABCC*) primarily governs pharmacokinetics and toxicity, whereas deep learning and network-based models have expanded hypotheses for its repurposing against emerging pathogens. Together, these approaches illustrate the dual contribution of pharmacogenomics and pattern recognition to precision antiviral development: defining the right patient population and refining the predictive tools that guide future clinical exploration.

### 4.5. AI-Assisted Drug Repurposing: Successes and Limitations

While ribavirin and lopinavir did not become cornerstone COVID-19 therapies, the pandemic nonetheless showcased notable successes of AI-assisted drug repurposing. As noted earlier, the case of baricitinib stands out as a proof-of-concept: an AI-derived hypothesis was validated in clinical trials and translated into clinical practice [5,32]. Another illustrative example is dexamethasone, a corticosteroid identified through conventional hypothesis and trial but retrospectively confirmed by computational analyses of clinical data using pattern mining to detect outcome patterns in patients receiving steroids. These instances demonstrate the promise of pattern recognition in rapidly sifting through vast possibilities compared to traditional methods.

At the same time, one must acknowledge the high proportion of predictions that did not materialize. Computational screens at various points nominated dozens of existing drugs—including antivirals such as favipiravir, antiparasitics such as ivermectin, and anti-diabetics such as metformin—for potential COVID-19 treatment. The majority either showed no clear benefit or produced inconclusive trial results. This high false-positive rate reflects multiple factors: computational models often simplify biological complexity, leading to optimistic assumptions that a compound inhibiting viral replication in a cell culture (or exhibiting in silico binding) will translate to clinical efficacy. Pattern recognition models may not account for pharmacokinetics, toxicity at the required doses, or disease stage (many antivirals are only effective if given very early, a nuance often missed in model outputs and in trial design).

The key lesson for future efforts is that pattern recognition algorithms should incorporate as many real-world constraints as possible. Recent proposals advocate for multifactorial models that combine viral inhibition data, immune modulation effects, pharmacokinetic modeling, and pharmacogenetic propensity into a composite “repurposing score” [6]. Other forward-looking approaches include reinforcement learning or multiobjective optimization to propose not only candidate drugs but also optimal dosing regimens or combinations, effectively learning a “policy” for treating a disease given patient-specific inputs.

On the pharmacogenomics side, the COVID-19 experience revealed another limitation: even when relevant genetic markers are known, implementing them in real time can be challenging. Health systems were not routinely genotyping patients for markers such as *IFNL3* or *CYP2C19* when treating COVID-19, as the immediate focus was on clinical care rather than stratification. This suggests that to fully exploit pattern recognition in emergencies, we need preemptive infrastructure—widespread genomic screening in populations and digital tools that can rapidly repurpose existing genomic knowledge for new threats. Conceptually, one can imagine an integrated system in which, upon the emergence of a new pathogen, its drug targets are cross-referenced with all known drug–gene interactions from other diseases and pattern-matched to identify which population subgroups (by genotype) might benefit from which existing drugs. Several proposals in the literature are moving in this direction, effectively merging pharmacogenomic databases with AI-driven decision support for emerging epidemics [6].

(1) Disadvantages and error profiles of AI-based repurposing.

The chief disadvantages stem from model–system mismatch and uncertainty under distribution shift. Docking scores and static protein–ligand descriptors incompletely capture conformational dynamics, active-site water, or allosteric regulation; transcriptomic signatures (e.g., connectivity mapping) may conflate causal and correlative signals; network-based proximity can overweigh highly connected hubs; and deep learning models trained on historical assays inherit batch effects and publication bias. As a result, false positives are expected—particularly when effect sizes in vitro require supra-physiologic concentrations or when PK/PD constraints (lung tissue exposure, protein binding, time above IC_50_) are not modeled. Errors also arise from label leakage, optimistic cross-validation on non-independent splits, and uncalibrated uncertainty, which together inflate apparent performance. In this domain, a nontrivial false-discovery rate is typical and should be anticipated; rigorous prospective validation, uncertainty calibration, and pre-registered analysis plans help contain it.

(2) Regulatory status and protocols toward a real drug substance.

No regulatory agency approves a new or repurposed drug *solely* on the basis of AI predictions. Standard pathways still apply: target and mechanism substantiation; preclinical pharmacology and toxicology; chemistry, manufacturing and controls (CMCs); an Investigational New Drug (IND) submission; and phased clinical trials to demonstrate safety and efficacy. For quality and manufacturing, ICH guidelines (e.g., Q8–Q12) govern process development and control; for clinical pharmacogenomics, implementation typically follows evidence-based guidance (e.g., CPIC) where available, but AI by itself is not a substitute for trial evidence. In emergencies, authorities may grant restricted pathways (e.g., emergency or conditional authorizations), yet these still require human data that address benefit–risk; AI can prioritize candidates, design trials, or support dosing hypotheses, but it does not replace statutory evidentiary standards.

(3) Outlook: how AI can yield more reliable results.

Reliability will improve by unifying mechanistic constraints with data-driven learning. Promising directions include the following: integrating PBPK/PD simulators with neural surrogates to enforce exposure–response realism; leveraging multimodal foundation models that jointly encode structure, sequence, omics, images, and EHR outcomes; adopting causal inference and target-trial emulation for robust real-world evidence; deploying active learning with prospective, uncertainty-driven assays; using calibrated ensembles and conformal prediction to quantify error; and shifting evaluation to prospective, pre-registered benchmarks with external replication. Federated learning and privacy-preserving analytics can unlock multi-institutional datasets, while adaptive platform trials provide rapid, feedback-coupled validation loops.

(4) Health risks associated with AI techniques.

Risks arise if models overstate efficacy or understate toxicity, potentially driving off-label use without adequate monitoring. Bias in training data can propagate inequities across demographic groups; spurious correlations may misguide prioritization; and opaque models can hinder clinician oversight. Cybersecurity and data-privacy breaches are additional concerns when integrating EHR and genomic data. Mitigations include human-in-the-loop governance, conservative decision thresholds, pharmacovigilance plans, dose-finding safeguards, equity audits, and model documentation (data sheets and model cards) that transparently communicate scope and limitations.

The broader discussion around successes like baricitinib vs. failures like lopinavir underscores that pattern recognition algorithms greatly accelerate hypothesis generation, but rigorous experimental validation remains the arbiter of success. The field is learning from each case to refine its models: for instance, by combining viral and host factors, by addressing model bias, and by incorporating uncertainty estimates so that predictions can be prioritized by confidence. The ultimate goal is a virtuous cycle where computational predictions inform trials, trials provide new data (e.g., about which subgroups responded), and those data feed back into improved models—a clear example of human–AI collaboration in drug discovery.

## 5. Conclusions and Future Work

Pattern recognition algorithms, including machine learning and deep learning techniques, have emerged as powerful allies in drug discovery and development. In pharmacogenomics, these methods have enabled the identification of genetic patterns (such as SNP signatures and biomarker profiles) that predict individual responses to medications, paving the way toward more personalized and effective therapy. In the realm of drug repurposing, computational pattern recognition has drastically cut down the search space for new treatments by intelligently sifting through existing drugs and biomedical knowledge to propose viable candidates, an approach that proved particularly valuable during the COVID-19 crisis. The case studies of ribavirin and lopinavir exemplify both the strengths and limitations of pattern recognition approaches in pandemic pharmacogenomics. We have seen that known pharmacogenomic markers for these antivirals (e.g., *IFNL3* and *ITPA* variants for ribavirin; transporter and enzyme polymorphisms for lopinavir) can be incorporated into predictive models to guide personalized therapy. However, the translation of these insights into the clinical management of COVID-19 was limited by the intrinsic efficacy of the drugs, as well as the availability and structure of supporting data. In contrast, the AI-guided prediction and subsequent clinical validation of baricitinib as an effective COVID-19 therapy highlight a successful case in which computational hypothesis generation aligned with empirical findings. The disparity between these outcomes underscores the need to understand not only how to identify promising patterns but also how to validate and integrate them into real-world care.

To consolidate the lessons drawn from this work and chart paths forward, we highlight five critical directions for future research and implementation:

1. Integration of multi-omics and clinical data: Pattern recognition models must evolve beyond single-modality inputs. A genomic signal may be informative, but its predictive power is amplified when integrated with other biological and clinical features. Multi-omics data—including transcriptomics, proteomics, and metabolomics—combined with real-world evidence such as electronic health records (EHRs), comorbidity profiles, and longitudinal treatment data, offer a more complete basis for inference. Emerging architectures in multimodal deep learning are well-suited to such integrative tasks. However, realizing this potential depends on coordinated data-sharing efforts, high-quality annotation standards, and robust ethical safeguards to protect patient privacy.
2. Algorithms for causality and interpretability: One key limitation of current AI approaches in pharmacogenomics and drug repurposing is their reliance on correlational patterns. Future models must incorporate causal reasoning to distinguish confounding from true biological effects. Furthermore, interpretability is not optional. Clinicians and regulators need to understand why a model recommends a certain drug for a given patient or condition. Causal inference methods (e.g., structural causal models and counterfactual analysis), interpretable ML techniques (e.g., attention visualization and SHAP values), and hybrid approaches that embed mechanistic biological knowledge (e.g., pathway-aware networks) are all promising directions to enhance the transparency and trustworthiness of pattern recognition in biomedicine.
3. Emphasis on combination therapies and complex patterns: Much of the existing work in both pharmacogenomics and repurposing has focused on single-drug, single-gene associations. Yet clinical reality often involves combination regimens and polygenic influences. Pattern recognition must account for drug–drug interactions, potential synergistic or antagonistic effects, and stratified patient responses. Deep learning frameworks for drug synergy prediction and tools to derive polygenic risk or response scores from large-scale genomic data are being actively developed and should be incorporated into future studies. The COVID-19 experience has illustrated the limitations of monotherapy and the potential of strategic combinations—such as antivirals paired with immunomodulators—identified via complementary pattern signals.
4. Real-time learning and adaptation: A major lesson from the pandemic is the need for adaptable models that update as new data arrive. Static predictions based on early assumptions may not hold as evidence evolves. Pattern recognition systems should be designed for continuous learning, ideally within federated learning architectures that respect data sovereignty. These systems can ingest updated clinical trial results, EHR data, and genomic findings to recalibrate drug prioritization, dosing strategies, or patient stratification recommendations. Such responsiveness enhances both the scientific robustness and the clinical utility of AI-guided pharmacotherapy.
5. Implementation and ethical considerations: As pattern recognition systems mature, implementation challenges will become paramount. Clinical decision support tools that integrate AI-derived predictions into prescribing workflows must be co-designed with end users to ensure usability and safety. Regulatory frameworks will need to assess not only the efficacy of drugs but also the validity of the algorithms used to select or personalize them. Ethically, special care is needed to avoid algorithmic bias. For example, pharmacogenomic patterns derived from a limited subset of populations may not generalize, potentially exacerbating health disparities. Diverse, representative training datasets and transparent reporting of model performance across subgroups are essential safeguards.

The present review has synthesized how pattern recognition algorithms—from classical machine learning to transformer and graph-based architectures—are reshaping pharmacogenomics and computational drug repurposing. By analyzing the paradigmatic cases of ribavirin and lopinavir, we have illustrated both the promise and the limitations of in silico approaches: they can efficiently reveal pharmacogenomic determinants and potential therapeutic hypotheses, yet they require rigorous experimental validation to translate predictions into clinically actionable insights.

To bridge this computational–experimental divide, we propose a clear roadmap for the next phase of translational research. This roadmap delineates how the hypotheses generated by computational pattern recognition can be progressively validated and refined through laboratory experimentation.

### 5.1. Preclinical Validation of Computational Hypotheses

The first step involves confirming the predicted drug–target and drug–gene associations derived from the models reviewed (e.g., MT-DTI, DeepPurpose, TxGNN). For each computationally inferred interaction,

- Conduct biochemical binding assays (e.g., surface plasmon resonance, fluorescence polarization, or differential scanning fluorimetry) to verify predicted affinities between ribavirin/lopinavir and their proposed viral or host targets.
- Perform molecular docking and dynamics simulations under controlled conditions to quantify binding stability, validate predicted Kd ranges, and assess allosteric or competitive effects suggested by the deep learning models.
- Use transcriptomic perturbation assays in human cell lines to confirm that predicted signature reversals (from CMap or LINCS data) correspond to actual gene expression changes upon drug exposure.

### 5.2. Pharmacogenomic Stratification In Vitro

A second level of validation requires evaluating the influence of genetic polymorphisms predicted to modulate response and toxicity:

- Establish CRISPR/Cas9-edited cell lines carrying specific variants such as *IFNL3* rs12979860, *ITPA* rs1127354/rs7270101, *SLCO1B1* rs4149056, and *ABCC2* rs717620 to reproduce interindividual variability.
- Assess differences in antiviral efficacy, cytotoxicity, and metabolite accumulation across genotypes under identical drug concentrations.
- Apply omics-level readouts (RNA-seq and metabolomics) to validate that genotype-dependent patterns predicted by machine learning correspond to real molecular phenotypes.

### 5.3. In Vivo Pharmacokinetic and Pharmacodynamic Modeling

Once in vitro findings confirm computational predictions, preclinical animal studies can close the next translational gap:

- Conduct pharmacokinetic (PK) profiling in humanized or transgenic models carrying orthologous polymorphisms of *ITPA* or *SLCO1B1*, evaluating differences in absorption, clearance, and toxicity.
- Integrate experimental PK/PD parameters into machine learning simulations to iteratively retrain predictive models, achieving bidirectional refinement between computation and biology.

### 5.4. Early-Phase Clinical Validation and Biomarker Translation

Ultimately, the hypotheses supported by both in silico and in vivo data should progress to small-scale clinical exploration:

- Design phase I–II genotype-stratified clinical trials for ribavirin or lopinavir combinations, recruiting participants based on their *IFNL3*, *ITPA*, or transporter genotypes.
- Collect and integrate multi-omics patient data (genomic, transcriptomic, metabolomic) into the same computational framework used in silico, closing the feedback loop between prediction and observation.
- Employ explainable AI techniques (e.g., SHAP and integrated gradients) to interpret patient-specific outcomes and refine biomarker selection for precision dosing.

### 5.5. Concluding Remarks

In conclusion, pattern recognition algorithms provide an unprecedented capability to map the vast chemical and genomic landscape underlying drug response. Yet, their true impact depends on how effectively we integrate them into the empirical workflow of drug development. The transition from prediction to verification—from algorithms to assays—will determine whether computational pharmacogenomics can fulfill its promise of accelerating safe and personalized therapies. The roadmap presented here aims to guide that transition, fostering a new generation of synergistic research where AI and laboratory science co-evolve toward precision medicine.

In conclusion, pattern recognition has demonstrated its power to accelerate and refine drug discovery, personalization, and repurposing. From the identification of actionable pharmacogenomic markers (such as *IFNL3* and *SLCO1B1*) to the AI-driven proposal of baricitinib as a COVID-19 therapy, we now stand at the threshold of a more systematic, integrative, and evidence-responsive approach to therapeutic innovation. The future lies in models that are not only accurate but also explainable, adaptable, and ethically grounded. Coupled with rigorous experimental validation and real-time feedback from clinical outcomes, such models could transform both how we develop drugs and how we deploy them—moving us closer to a paradigm of anticipatory, precision-guided medicine in future pandemics and beyond.

## Figures and Tables

**Table 1:** Computational drug repurposing approaches used in COVID-19.

Approach	Data Type	Strengths	Limitations	Refs.
Knowledge graphs/ network mining	Drug–target, PPI, pathway graphs	Mechanistic paths; explainability; rapid hypothesis generation	Knowledge bias; incomplete graphs	[4,5]
Deep DTI/ chemogenomics	Molecular structures; target features	Learns complex nonlinear drug–target patterns	Data hungry; limited PK/PD context	[23,29]
Signature reversal (transcriptomics)	Drug/cell expression signatures	Matches drug-induced changes against disease signatures	Sensitive to batch effects and biological context	[30,31]
Executable/ systems networks	Virus–host causal models	Combination prediction; mechanistic insight	Heavy modeling effort; calibration needed	[4]

**Table 2:** Pattern recognition and machine learning techniques used in pharmacogenomics.

Technique	Task	Inputs	Typical Use in PGx	Refs.
SVM	Classification	SNPs; clinical covariates	Predict response to IFN + RBV	[1,3,14]
Random Forest	Feature selection	SNPs; gene expression	Biomarker ranking (variable importance)	[15]
Penalized GLM (LASSO)	Sparse modeling	High-dim. SNPs/omics	Compact predictive signatures	[14]
ANN/CNN/RNN	Representation learning	Sequences; multi-omics	Nonlinear patterns; sequence models	[2,12,42]
Haplotype analysis + pattern recognition	Association/cls.	Phased SNPs	Haplotype-aware predictors	[11]
GWAS + ML	Hybrid discovery	Genome-wide SNPs	From association to prediction	[14]

**Table 3:** Key pharmacogenomic variants for ribavirin (RBV) and lopinavir (LPV).

Drug	Gene	Variant (rsID)	Reported Effect	Refs.
RBV	*IFNL3*	rs12979860	Higher SVR with CC genotype in HCV; inferred relevance to RBV-based regimens	[13,49,50]
RBV	*ITPA*	rs1127354, rs7270101	Reduced risk of RBV-induced hemolytic anemia (ITPase deficiency)	[13,51]
LPV	*SLCO1B1*	rs11045819 (*4)	Increased LPV clearance (lower exposure)	[52]
LPV	*SLCO1B1*	rs4149056 (*5)	Decreased LPV clearance (higher exposure)	[52]
LPV	*ABCC2*	rs717620	Transport variation associated with PK differences	[52]

## Data Availability

No new data were created or analyzed in this study. Data sharing is not applicable to this article.

## Abbreviations

The following abbreviations are used in this manuscript:

AI	Artificial Intelligence
ABCC2	ATP-Binding Cassette Subfamily C Member 2 (MRP2)
ANN	Artificial Neural Network
AUC	Area Under the Curve
AUPRC	Area Under the Precision–Recall Curve
CC	Cytokine Cluster (context: genotype CC for IFNL3 rs12979860)
CNN	Convolutional Neural Network
CPI	Compound–Protein Interaction
COVID-19	Coronavirus Disease 2019
DL	Deep Learning
DTI	Drug–Target Interaction
GNN	Graph Neural Network
GWAS	Genome-Wide Association Study/Studies
IFNL3	Interferon Lambda 3
ITPA	Inosine Triphosphatase
Kd	Dissociation Constant (binding affinity)
LASSO	Least Absolute Shrinkage and Selection Operator
LINCS	Library of Integrated Network-Based Cellular Signatures
LPV	Lopinavir
ML	Machine Learning
MPXV	Monkeypox Virus
mRMR	minimum Redundancy Maximum Relevance
MT-DTI	Molecule Transformer–Drug–Target Interaction
PPI	Protein–Protein Interaction
PGx	Pharmacogenomics
PK	Pharmacokinetics
PD	Pharmacodynamics
RBV	Ribavirin
RF	Random Forest
RNN	Recurrent Neural Network
SARS-CoV-2	Severe Acute Respiratory Syndrome Coronavirus 2
SLC28A1/A2/A3	Solute Carrier Family 28 Members (Nucleoside Transporters)
SLC29A1	Solute Carrier Family 29 Member 1 (ENT1 Transporter)
SLCO1B1	Solute Carrier Organic Anion Transporter Family Member 1B1 (OATP1B1)
SNP	Single-Nucleotide Polymorphism
SVM	Support Vector Machine
TxGNN	Therapeutic Graph Neural Network
VCTatMLP	Virus–Compound–Target attentional Multi-Layer Perceptron
VCTatDot	Virus–Compound–Target attentional Dot-Product Model
